# Supplementary material for: SARS-CoV-2 spike glycoprotein vaccine candidate NVX-CoV2373 immunogenicity in baboons and protection in mice
Source: Nat Commun. 2021 Jan 14;12:372. doi: 10.1038/s41467-020-20653-8 (PMC7809486; doi:10.1038/s41467-020-20653-8)
Supplement: Supplementary file 1 — Supplementary Information [file 41467_2020_20653_MOESM1_ESM.pdf]

Supplementary Information for:

SARS-CoV-2 spike glycoprotein vaccine candidate NVX-CoV2373 immunogenicity in baboons and protection in mice

Jing-Hui Tian[1 #], Nita Patel[1 #], Robert Haupt[2 #], Haixia Zhou[1], Stuart Weston[2], Holly Hammond[2], James Logue[2], Alyse D. Portnoff[1], James Norton[1], Mimi Guebre-Xabier[1], Bin Zhou[1], Kelsey Jacobson[1], Sonia Maciejewski[1], Rafia Khatoon[1], Malgorzata Wisniewska[1], Will Moffitt[1], Stefanie Kluepfel-Stahl[1], Betty Ekechukwu[1], James Papin[3], Sarathi Boddapati[4], C. Jason Wong[4], Pedro A. Piedra[5], Matthew B. Frieman[2], Michael J. Massare[1], Louis Fries[1], Karin Lövgren Bengtsson[6], Linda Stertman[6], Larry Ellingsworth[1], Gregory Glenn[1], and Gale Smith[1 *]

[1]Novavax, Inc. 21 Firstfield Road, Gaithersburg, MD, 20878, USA.

[2]University of Maryland, School of Medicine, 685 West Baltimore St, Baltimore, MD 21201, USA.

[3]University of Oklahoma, Health Sciences Center, Department of Pathology, Division of Comparative Medicine, 940 Stanton L. Young, BMS 203, Oklahoma City, OK, 73104 USA.

[4]Catalent Cell & Gene Therapy, 20 Firstfield Road, Gaithersburg, MD, 20874, USA.

[5]Department of Molecular Virology and Microbiology, and Pediatrics, Baylor College of Medicine, Houston, Texas.

[6]Novavax AB, Kungsgatan 109, Uppsala, SE-753 18, SE.

[#]These authors contributed equally.

*Correspondence: GSmith@Novavax.com.

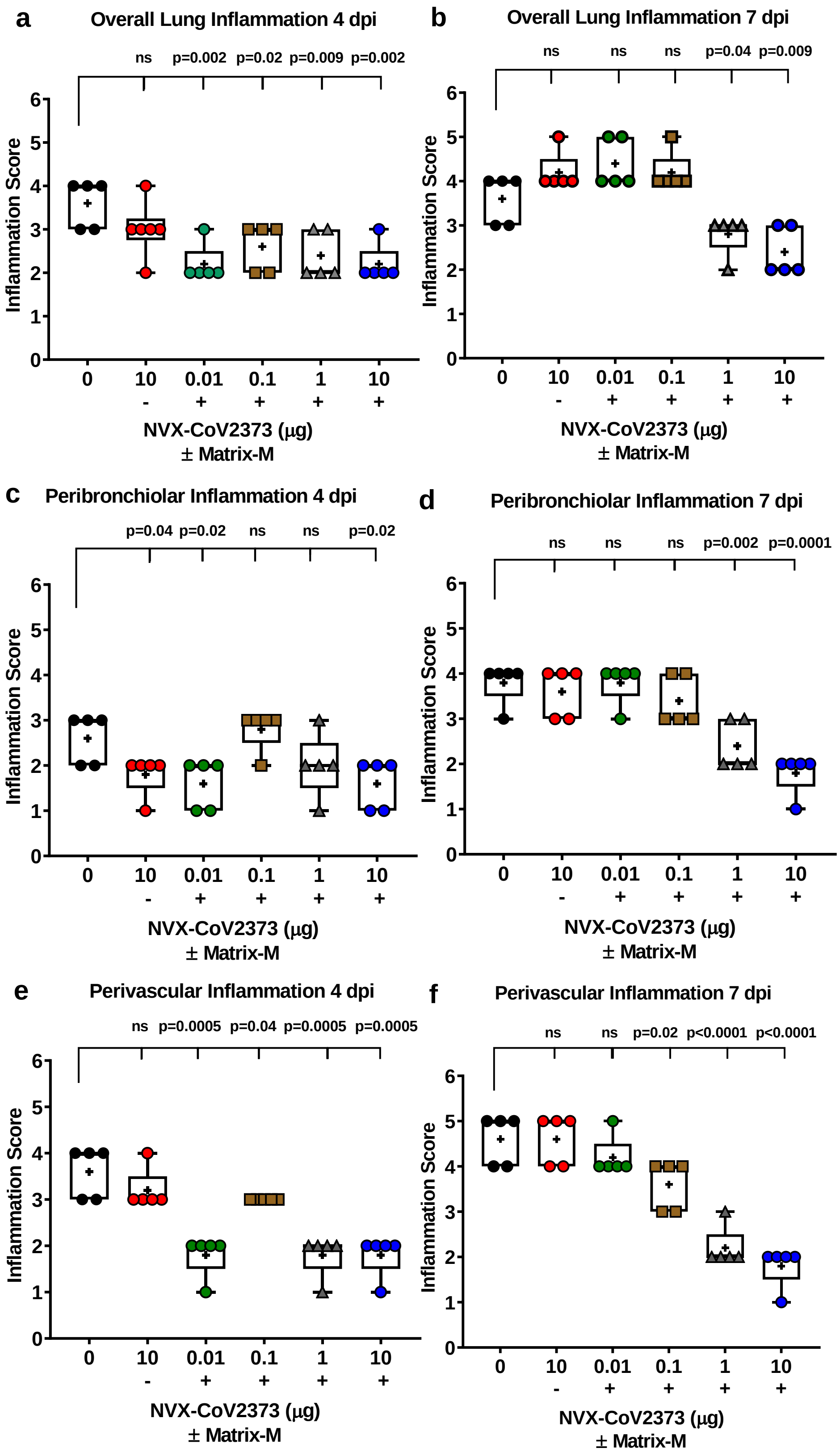
a
Overall Lung Inflammation 4 dpi
ns p=0.002 p=0.02 p=0.009 p=0.002
Inflammation Score
0 10 0.01 0.1 1 10
- + + + +
NVX-CoV2373 (μg)
± Matrix-M
b
Overall Lung Inflammation 7 dpi
ns ns ns p=0.04 p=0.009
Inflammation Score
0 10 0.01 0.1 1 10
- + + + +
NVX-CoV2373 (μg)
± Matrix-M
c
Peribronchiolar Inflammation 4 dpi
p=0.04 p=0.02 ns ns p=0.02
Inflammation Score
0 10 0.01 0.1 1 10
- + + + +
NVX-CoV2373 (μg)
± Matrix-M
d
Peribronchiolar Inflammation 7 dpi
ns ns ns p=0.002 p=0.0001
Inflammation Score
0 10 0.01 0.1 1 10
- + + + +
NVX-CoV2373 (μg)
± Matrix-M
e
Perivascular Inflammation 4 dpi
ns p=0.0005 p=0.04 p=0.0005 p=0.0005
Inflammation Score
0 10 0.01 0.1 1 10
- + + + +
NVX-CoV2373 (μg)
± Matrix-M
f
Perivascular Inflammation 7 dpi
ns ns p=0.02 p<0.0001 p<0.0001
Inflammation Score
0 10 0.01 0.1 1 10
- + + + +
NVX-CoV2373 (μg)
± Matrix-M

Supplementary Figure 1. Histopathology scores of NVX-CoV2373 immunized mice challenged with SARS-CoV-2. Groups of mice (N = 10/group) were immunized twice (day 0 and 14) with NVX-CoV2373 with or without Matrix-M (5 µg). The placebo group received formulation buffer. Following vaccination, mice were made permissive to SARS-CoV-2 infection by intranasal (IN) transduction with 2.5 x 10^8 pfu Ad/hACE2, 38 days after immunization. At four days post transduction, mice were challenged intranasal with 1.5 x 10^5 pfu of SARS-CoV-2. Five mice from each group were sacrificed at 4 days (**a, c,** and **e**) and 7 days (**b, d,** and **f**) post infection (dpi). Lung tissues were collected, fixed, paraffin embedded and sections stained with hematoxylin and eosin. Sections were scored on a severity scale of 0-5. In the box-and-whisker plots, the mean is indicated by plus, the top and bottom of the box is the 25th and 75th percentile, and the whiskers the minima and maxima range. Symbols represent individual animal scores. Student's t-test (unpaired, two tail) was used to compare significant differences in vaccinated groups compared to the placebo group. Not significant (ns).

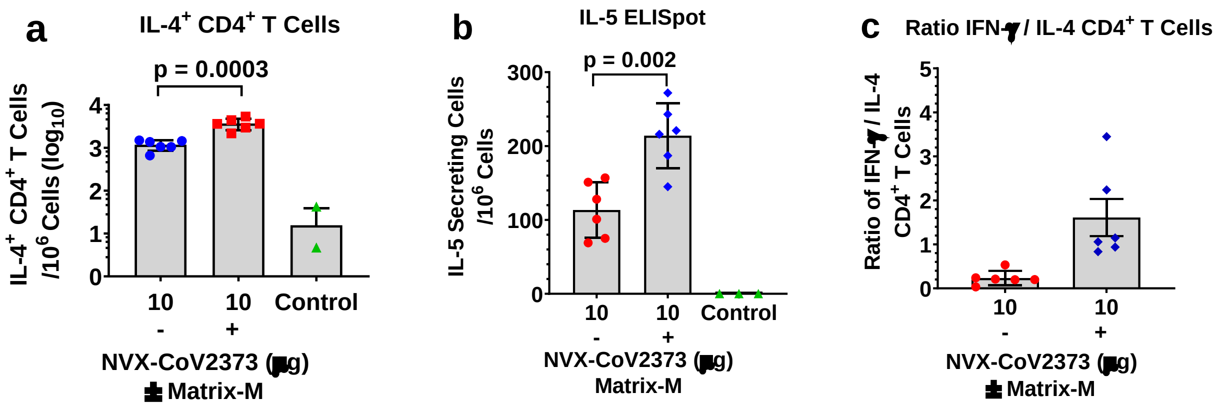

Supplementary Figure 2. Intracellular staining (ICCS) and ELISpot detection of type 2 cytokines in immunized mice. Groups of mice (n = 6/group) were immunized with NVX-CoV2373 with and without 5 µg Matrix-M adjuvant with 2 doses spaced 21 days apart and splenocytes collected 7-days after the second immunization and stimulated with NVX-CoV2373 protein. (**A**) ICCS of IL-4^+ $CD4^+$ T cells. (**B**) IL-5-secreting splenocytes determined by ELISpot analysis. (**C**) Ratio of antigen-specific IFN-γ^+ to IL-4^+ $CD4^+$ T cells in spleens of mice immunized with NVX-CoV2373 with and without Matrix-M adjuvant. The mean is indicated by bars and error bars indicate the ±SD and individual animal values are indicated by the colored symbols. Student's t-test (unpaired, two tail) was used to determine significant differences between groups receiving the adjuvanted NVX-CoV2373 compared to animals receiving the non-adjuvanted vaccine.

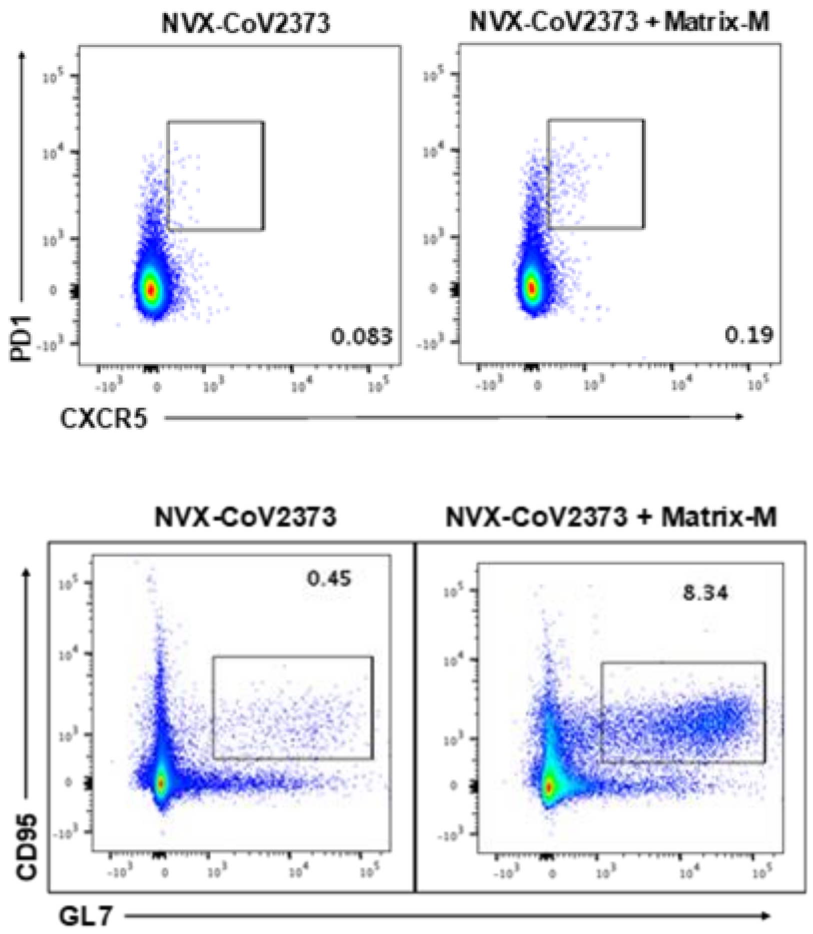

Supplementary Figure 3. Gating strategy used to determine frequencies of follicular helper T cells (Tfh) and germinal center B cells (GC) generated by immunization with NVX-CoV2373 and Matrix-M adjuvant in mice. Stimulated cells were stained and analyzed by flow cytometry. The samples were gated to identify single live cells.
