# Supplementary material for: SARS-CoV-2 spike glycoprotein vaccine candidate NVX-CoV2373 immunogenicity in baboons and protection in mice
Source: Nat Commun. 2021 Jan 14;12:372. doi: 10.1038/s41467-020-20653-8 (PMC7809486; doi:10.1038/s41467-020-20653-8)
Supplement: Supplementary file 3 — Reporting Summary [file 41467_2020_20653_MOESM3_ESM.pdf]

nature research

Corresponding author(s): Gale Smith
Last updated by author(s): Oct 20, 2020

Reporting Summary

Nature Research wishes to improve the reproducibility of the work that we publish. This form provides structure for consistency and transparency in reporting. For further information on Nature Research policies, see our Editorial Policies and the Editorial Policy Checklist.

Statistics

For all statistical analyses, confirm that the following items are present in the figure legend, table legend, main text, or Methods section.

n/a	Confirmed	
☐	☒	The exact sample size (*n*) for each experimental group/condition, given as a discrete number and unit of measurement
☐	☒	A statement on whether measurements were taken from distinct samples or whether the same sample was measured repeatedly
☐	☒	The statistical test(s) used AND whether they are one- or two-sided<br>*Only common tests should be described solely by name; describe more complex techniques in the Methods section.*
☒	☐	A description of all covariates tested
☒	☐	A description of any assumptions or corrections, such as tests of normality and adjustment for multiple comparisons
☐	☒	A full description of the statistical parameters including central tendency (e.g. means) or other basic estimates (e.g. regression coefficient) AND variation (e.g. standard deviation) or associated estimates of uncertainty (e.g. confidence intervals)
☐	☒	For null hypothesis testing, the test statistic (e.g. F, t, r) with confidence intervals, effect sizes, degrees of freedom and P value noted<br>*Give P values as exact values whenever suitable.*
☒	☐	For Bayesian analysis, information on the choice of priors and Markov chain Monte Carlo settings
☒	☐	For hierarchical and complex designs, identification of the appropriate level for tests and full reporting of outcomes
☒	☐	Estimates of effect sizes (e.g. Cohen's d, Pearson's r), indicating how they were calculated

Our web collection on statistics for biologists contains articles on many of the points above.

Software and code

Policy information about availability of computer code

Data collection	GraphPad Prism version 7.05, Zetasizer NanopZS solfware v7.12, NanoDSC v4.6.0 (Mettler Toledo), XMIPP processing package for 2D images, Octet software HT 101:1, Softwax Pro 6.5.1 GxP software, ImmunoSpot software v5.03, FlowJo software version Xv10
Data analysis	This study used commercially available GraphPad Prism software v7.05 for data presentation and statistical analysis. For intracellular cytokine analysis, all stained samples were acuired using LSR Fortess flow cytometer (Becton Dickinson) and data were analyzed with FlowJo software version Xv10 (Tree Star, Inc). For ELISA methods, a SpectraMax Plus plate reader was used and data analyzed with SoftMax software (Molecular Devices). For TEM and 2 D class averaging a FEI Tecani T12 electron microscope equipped with a FEI Eagle 4k x 4k CCD camera was used and XMIPP processing package (Scripion, Inc) used to for particle alignment and classification. For bio-layer interferometry (BLI) analysis an Octet QK384 system (Pall ForteBio) was used and data analyzed with Octet software HT (Pall ForteBio).

For manuscripts utilizing custom algorithms or software that are central to the research but not yet described in published literature, software must be made available to editors and reviewers. We strongly encourage code deposition in a community repository (e.g. GitHub). See the Nature Research guidelines for submitting code & software for further information.

Data

Policy information about availability of data

All manuscripts must include a data availability statement. This statement should provide the following information, where applicable:
- Accession codes, unique identifiers, or web links for publicly available datasets
- A list of figures that have associated raw data
- A description of any restrictions on data availability

All data generated or analyzed during this study are included in this published article and supplementary information files.

Field-specific reporting

Please select the one below that is the best fit for your research. If you are not sure, read the appropriate sections before making your selection.

☒ Life sciences ☐ Behavioural & social sciences ☐ Ecological, evolutionary & environmental sciences

For a reference copy of the document with all sections, see nature.com/documents/nr-reporting-summary-flat.pdf

Life sciences study design

All studies must disclose on these points even when the disclosure is negative.

Sample size	No sample size calculations were used to power each study. Sample size for the mouse studies were determined based upon previous experience with similar studies where 5-10 animals per group represented a sufficient sample size (Coleman, C. et al. Vaccine, 2017, 35;1586; Coleman, C et al. Vaccine, 2014, 32;3169; Liu, Y. et al. Vaccine, 2011, 29;6606). Sample size was also limited by space available in a ABSL-3 containment facility. To assess reproducibility, groups of 10 mice were immunized over a dose range (0.01-10 ug) of NVX-CoV2373 vaccine to assess the dose effect. The baboon was used as a second model for assessing the humoral and cellular immune responses to NVX-Co2373 with the Matrix-M adjuvant. The sample size was based upon availability of healthy adult male and female baboons. The baboon study is intended to compare antibody levels in immunized animals to antibodies levels in serum from patients recovering from recent SARS-CoV-2 infection.
Data exclusions	No data were excluded.
Replication	Replicate samples were performed for serological assessments. In addition, reproducibility was determined in groups of 10 mice immunized over wide dose range (0.01, 0.1, 1.0 and 10 ug) of NVX-CoV2373. Replicates were reproducible and none failed. Data points represent the mean values of two technical replicates as stated in the figure legend.
Randomization	For mouse and baboon studies, animals were randomly assigned to vaccination groups.
Blinding	For histological and clinical scoring, the histologist and technician was blind to the study groups. Other experiments involving immunological evaluation, analysis of serum samples was performed by an analyst who was blind to the immunization group.

Reporting for specific materials, systems and methods

We require information from authors about some types of materials, experimental systems and methods used in many studies. Here, indicate whether each material, system or method listed is relevant to your study. If you are not sure if a list item applies to your research, read the appropriate section before selecting a response.

Materials & experimental systems

n/a	Involved in the study	
☐	☒	Antibodies
☐	☒	Eukaryotic cell lines
☒	☐	Palaeontology and archaeology
☐	☒	Animals and other organisms
☒	☐	Human research participants
☒	☐	Clinical data
☒	☐	Dual use research of concern

Methods

n/a	Involved in the study	
☒	☐	ChIP-seq
☐	☒	Flow cytometry
☒	☐	MRI-based neuroimaging

Antibodies

Antibodies used

ELISA:
HRP-goat anti-mouse IgG (Southern Biotech, 1036-05)
HRP-goat anti-human IgG (Southern Biotech, 2015-05)
hACE2 receptor ELISA:
Rabbit anti-SARS-CoV spike protein (BEI Resources, NR-4569)
HRP-conjugated mouse anti-histidine IgG (Southern Biotech, 4603-05)

ELISpot:
Mouse IFN-gamma ELISpot basic kit (HRP) (MabTech, 3321-2H)
Mouse IL-5 ELISpot basic kit (HRP) (MabTech, 3391-2H).

Intracellular cytokine staining (ICC):

CD3 BV650 (BD, 563851)

CD4 APC-H7 (BD, 560246)
CD8 FITC (BD, 553030)
PD-1 APC (BD, 562671)
CXCR5 Percp-Cy5.5 (BD, 560528)
CD19 AF700 (BD, 557958)
CD49b PE (BD, 558759)
CD3 FITC (BD, 553062)
CD19 APC (BD, 550992)
CD95 PE-cy7 (BD, 557653)
GL7 BV421 (BD, 561530)
B220 Percp-Cy5.5 (BD, 552771)
CD44 AF700 (BD, 560567)
CD62L PE (BD, 561918)
IL-2 BV421 (BD, 562969)
IFN-γ PerCP-Cy5.5 (BD, 560660)
TNF-α PE-Cy7 (BD, 557644)
IL-4 APC (BD 554436

Validation

ELISA and ELISPOT:
Antibody reagents are commercially available. Antibodies used in specific species have been appropriately validated by manufacturers and is information is provided on their website and data sheets as follows:
Rabbit anti-SARS-CoV spike protein cat. no. NR-4569 (https://www.beiresources.org/).
HRP goat anti-mouse IgG cat. no. 1036-05 (https://www.southernbiotech.com/).
HRP goat anti-human IgG cat. no. 2015-05 (https://www.southernbiotech.com/),
HRP mouse anti-histidine IgG cat. no. 4603-05 (https://www.southernbiotech.com/)
Mouse and human IFN-gamma, IL-5 and IL-4 ELISpot (https://www.mabtech.com/)

ICCS:
BV650 Mouse anti-human CD3 (BD, 563851). Ref to publication, PMID: 6788570
APC-H7 Rat anti-mouse CD4 (BD, 560246). Ref to publication, PMID: 2653377
FITC Rat anti-mouse CD8a (BD, 553030), Ref to publication, PMID: 2653377
APC Hamster anti-mouse CD279 PD-1 (BD, 562671), Ref to publication, PMID: 8671665
PerCP-Cy™5.5 Rat Anti-Mouse CD185 (CXCR5) (BD, 560528), Ref to publication, PMID: 7639692
Purified mouse anti-human CD19 AF700 (BD, 557958), Ref to publication, PMID: 7542548
PE Hamster anti-moused CD49b (BD, 558759), Ref to publication, PMID: 11466327
FTIC Hamster anti-mouse CD3 (BD, 553062), Ref to publication, Duke, RC et al. Current Protocols in Immunology. New York: John Wiley and Sons; 1995; :3.17.1-3.17.33.
APC Rat anti-mouse CD19 (BD, 550992), Ref to publication, PMID: 7542548
PE-Cy™7 Hamster Anti-Mouse CD95 BD, 557653), Ref to publication, PMID: 7523140
PE Rat Anti-Mouse T- and B-Cell Activation Antigen 30GL7 BV421 (BD, 561530), Ref to publication, PMID: 9323211
PerCP-Cy™5.5 Rat Anti-Mouse CD45R/B220 (BD, 552771), Ref to publication, PMID: 1383316
Alexa Fluor® 700 Rat Anti-Mouse CD44 (BD, 560567), Ref to publication, PMID: 7546402
PE Rat Anti-Mouse CD62L (BD, 561918), Ref to publication, PMID: 9647212
BV421 Rat Anti-Mouse IL-2 (BD, 562969), Ref to publication, PMID: 18432811
PerCP-Cy™5.5 Rat Anti-Mouse IFN-γ (BD, 560660), Ref to publication, PMID: 2960769
PE-Cy™7 Rat Anti-Mouse TNF-α PE-Cy7 (BD, 557644), Ref to publication, PMID: 1387110
APC Rat Anti-Mouse IL-4 (BD 554436), Ref to publication, PMID: 18432811

Eukaryotic cell lines

Policy information about cell lines

Cell line source(s)	VERO E6 cells were obtained from ATCC, CRL-1586; Sf9 insect cells obtained from ATCC, CRL-1711
Authentication	Vendor certificate of analysis was used to verify.
Mycoplasma contamination	VERO E6 cells were tested by ATCC and were free of mycoplasma. Sf9 cells were obtained from a validated working cell bank that was tested for adventitous agents including mycoplasma.
Commonly misidentified lines (See ICLAC register)	No commonly misidentified cell lines were used in this study.

Animals and other organisms

Policy information about studies involving animals; ARRIVE guidelines recommended for reporting animal research

Laboratory animals

The immunization phase of the mouse studies were performed by Noble Life Sciences (Syeville, MD) female BALB/c mice (7-9 weeks old, 17-22 grams) were randomly assigned to groups of 7-10 animals and housed in groups of up to 5 mice per cage. Ambient

	temperature (18-24C) and 40-60% relative humidity was continuously monitored. Mice were fed Purina mouse chow and maintained on a 12 hour cycle with lights on and off. Animals were fed and provided filtered water ad libitum.<br><br>For the SARS-CoV-2 challenge phase of the study, immunized mice were transferredfrom Noble Life Sciences to the University of Maryland BSL-3 containment facility. Animals were housed in groups up to 5 mice/cage and Purine mouse chow. Mice wer housed at ambient temperatures (18-24C) with a relative humidity of 40-60% with 12 hours of lights on and off each day.<br><br>Ten adult male and female (10-16 years old) olive baboons (Papio cynocephalus Anubis) were housed at the University of Oklahoma Health Sciences Center (OUHSC). Animals were housed indoors in accordance with OUHSC Institutional Biosafety Committee guidelines. Animals were randomly assigned to groups of 2-3/group and housed in individual cages.
Wild animals	The study did not involve wild animals.
Field-collected samples	The study did not involve field-collected samples
Ethics oversight	The mouse immunogenicity studies were performed by Noble Life Sciences (Sykeville, MD). Noble Life Sciences is accredited by the Association for Assessment and Accreditation of Laboratory Animal Care (AAALACC International). The mouse SARS-CoV-2 challenge study was conducted at the University of Maryland BSL-3 containment facility (College Park, MD). All animal procedures were in accordance with NRC Guide for the Care and Use of Laboratory Animals, the Animal Welfare Act, and the CDC/NIH Biosafety in Microbiological and Biomedical Laboratories. The olive baboon (Papio cynocephalus anubis) study was performed at the University of Oklahoma Health Science Center (OUHSC). OUHSC is accredited by AAALACC International. Baboons were maintained and treated according to the Institutional Biosafety Committee guidelines. Baboon experiments were approved by the Institutional Animal Care and Use Committee (IACUC) and the Institutional Biosafety Committee of OUHSC. Studies were conducted in accordance with the National Institutes of Health Guide for Care and Use of Laboratory Animals (NIH publication 8023, Revised 1978).

Note that full information on the approval of the study protocol must also be provided in the manuscript.

Flow Cytometry

Plots

Confirm that:

- [x] The axis labels state the marker and fluorochrome used (e.g. CD4-FITC).
- [x] The axis scales are clearly visible. Include numbers along axes only for bottom left plot of group (a 'group' is an analysis of identical markers).
- [x] All plots are contour plots with outliers or pseudocolor plots.
- [x] A numerical value for number of cells or percentage (with statistics) is provided.

Methodology

Sample preparation	Mouse splenocytes were washed with RPMI with 5% FBS, and red blood cells were lysed with ACK lysis buffer. Samples were resuspended in RPMI with 5% FBS for stimulation, and prepared in FACS buffer for Ab staining. NHP PBMC were processed using BD CPT tube, and then cryopreserved before shipping to Novavax. Frozen PBMC were thawed and cultured in RPMI with 10% FBS. Cells were prepared in FACS buffer for Ab staining.
Instrument	BD Fortessa
Software	Diva to process samples on BD Fortessa. Flowjo xV10 were used to analyze the data.
Cell population abundance	N/A. No sorting was performed
Gating strategy	FSC/SSC gate is applied for lymphocyte gate, FSC-A and FSC-H to gate on single cell population, Live dye negative population for live cells. The cells were further gated on CD3+ T lymphocytes, and then CD4 and CD8 to gate on CD4+ T cells. All these cell population was distinct cell population with clear pattern to distinguish positive and negative populations. The cytokine producing cells were gated based on medium only control and FMO.

- [x] Tick this box to confirm that a figure exemplifying the gating strategy is provided in the Supplementary Information.
